# The effects of green tea supplementation on cardiovascular risk factors: A systematic review and meta-analysis

**DOI:** 10.3389/fnut.2022.1084455

**Published:** 2023-01-10

**Authors:** Mohammad Zamani, Mahnaz Rezaei Kelishadi, Damoon Ashtary-Larky, Niusha Amirani, Kian Goudarzi, Iman Attackpour Torki, Reza Bagheri, Matin Ghanavati, Omid Asbaghi

**Affiliations:** ^1^Department of Clinical Nutrition, School of Nutritional Sciences and Dietetics, Tehran University of Medical Sciences, Tehran, Iran; ^2^Department of Community Nutrition, School of Nutrition and Food Science, Isfahan University of Medical Sciences, Isfahan, Iran; ^3^Nutrition and Metabolic Diseases Research Center, Ahvaz Jundishapur University of Medical Sciences, Ahvaz, Iran; ^4^Faculty of Medicine, Alborz University of Medical Sciences, Tehran, Iran; ^5^Faculty of Medicine, Shahid Beheshti University of Medical Sciences, Tehran, Iran; ^6^Faculty of Medicine, Golestan University of Medical Sciences, Gorgan, Iran; ^7^Department of Exercise Physiology, University of Isfahan, Isfahan, Iran; ^8^National Nutrition and Food Technology Research Institute, Faculty of Nutrition Sciences and Food Technology, Shahid Beheshti University of Medical Sciences, Tehran, Iran; ^9^Cancer Research Center, Shahid Beheshti University of Medical Sciences, Tehran, Iran; ^10^Student Research Committee, Shahid Beheshti University of Medical Sciences, Tehran, Iran

**Keywords:** green tea supplementation, cardiovascular risk factors, systematic review, meta-analysis, lipid profile, glycemic control, blood pressure

## Abstract

**Purpose:**

A bulk of observational studies have revealed the protective role of green tea supplementation in cardiovascular diseases. The current systematic review and meta-analysis study aimed to establish the effects of green tea supplementation on cardiovascular risk factors including lipid profile, blood pressure, glycemic control markers and CRP.

**Methods:**

A systematic literature search of randomized clinical trials (RCTs) that investigated the effects of green tea supplementation and cardiovascular risk factors was undertaken in online databases including PubMed/Medline, Scopus, Web of Science, and Embase using a combination of green tea and cardiovascular risk factors search terms. Meta-analyses were carried out using a random-effects model. The I^2^ index was used to assess the heterogeneity of RCTs.

**Results:**

Among the initial 11,286 studies that were identified from electronic databases search, 55 eligible RCTs with 63 effect sizes were eligible. Results from the random effects meta-analysis showed that GTE supplementation significantly reduced TC (WMD = −7.62; 95% CI: −10.51, −4.73; *P* = < 0.001), LDL-C (WMD = −5.80; 95% CI: −8.30, −3.30; *P* = < 0.001), FBS (WMD = −1.67; 95% CI: −2.58, −0.75; *P* = < 0.001), HbA1c (WMD = −0.15; 95% CI: −0.26, −0.04; *P* = 0.008), DBP (WMD = −0.87; 95% CI: −1.45, −0.29; *P* = 0.003), while increasing HDL-C (WMD = 1.85; 95% CI: 0.87, 2.84; *P* = 0.010). Subgroup analyses based on the duration of supplementation (≥ 12 *vs.* < 12 weeks), dose of green tea extract (GTE) (≥1,000 *vs.* < 1,000 mg/d), sex (male, female, and both), baseline serum levels of lipid profile, and glycemic control factors demonstrated different results for some risk factors.

**Conclusion:**

The current study suggests improvements in the lipid and glycemic profiles following green tea supplementation. These findings support previous evidence showing the health benefits of green tea supplementation on cardiometabolic risk factors.

## Introduction

Many people have considered green tea as a drink with health-promotion properties ranging from weight management to cancer prevention (1). Green tea extract (GTE) is a dietary supplement derived from *Camellia sinensis* leaves (2). To stop the fermentation process which reduces the polyphenols content of tea, freshly green tea leaves are steamed immediately upon harvest (3). The fact that GTE contains a large number of concentrated components, including non-oxidized polyphenols, vitamins, and antioxidants, is the basis for their current rise in popularity. The major phenolic compounds found in green tea are flavonoids accounting for nearly 70% of its total polyphenols (4). Catechins and their derivatives especially epigallocatechin-3-gallate (EGCG) are the most abundant flavonoids in green tea which are responsible for potential preventive effects of green tea on oxidative stress-caused diseases such as cancer, cardiovascular and neurodegenerative diseases (5).

Globally, cardiovascular diseases (CVD) continue to be the leading cause of death (6). Observational studies have suggested the primary preventive role of green tea against CVD such as stroke, coronary heart disease, and coronary atherosclerosis (7–9). In this regard, results from a large cohort study showed that daily consumption of 2 cups of green tea was associated with a 22-33% reduction in CVD-cause mortality among the Japanese population (10). Accumulating evidence has examined the effects of green tea products on traditional and novel cardiovascular risk factors such as hypertension, lipid disorders, diabetes, oxidative stress, endothelial dysfunction, and inflammation (11). Among lifestyle modification strategies for controlling CVD risk factors, regular consumption of functional foods rich in antioxidants and polyphenols such as coffee (12), dark chocolate (13), nuts (14) and green tea (12) have been proposed to promote cardiometabolic risk factors.

Although many factors play a pathogenic role, increased oxidative stress is a common potential cause of various CVD (15). The bulk of evidence has shown that the cardio-protective activity of green tea is mainly attributed to the antioxidant properties of its catechins which act by inducing anti-oxidant enzymes, inhibiting pro-oxidant enzymes, and scavenging free radicals (16, 17). In line with animal studies where green tea catechins had lowering effects on cholesterol (18, 19), the administration of green tea catechins has been reported to reduce total cholesterol (TC) and low-density lipoprotein (LDL) in human clinical trial studies (20). Although the exact mechanism of action of green tea to reduce cholesterol is not fully understood, an increase in thermogenesis, enhance gene expression of enzymes involved in bile acid production and appetite suppression has been proposed as potential mechanisms (21). Also, the supplementation with GTE with a high amount of catechins exerted favorable effects on glycemic control (22) and blood pressure (23). However, inconsistency between the results of recent studies has been identified regarding the effects of green tea supplementation on some CVD risk factors. For instance, 3-week high doses of green tea polyphenols supplementation failed to improve CVD risk factors except for TC: high-density lipoprotein (HDL) ratio among healthy men (24). Likewise, Mousavi et al. (25) did not report a significant reduction in TC, triglyceride (TG), LDL, or glycemic control markers in diabetic patients following the 8-week drinking of four cups of green tea compared to the control group. Owing to this consistency across clinical trial studies, the main objective of this systematic review and meta-analysis was to summarize the effects of green tea supplementation on cardiovascular risk factors including glycemic control markers (fasting blood sugar (FBS), hemoglobin A1C (HbA1c), homeostatic model assessment for insulin resistance (HOMA-IR), fasting insulin), blood pressure (systolic blood pressure [SBP] and diastolic blood pressure [DBP]) lipid profile (TG, TC, LDL, HDL) and C-reactive protein (CRP).

## Materials and methods

### Search strategy

Guidelines of the Preferred Reporting Items for Systematic Reviews and Meta-analysis (PRISMA) were considered in the current review. Data were searched in PubMed/MEDLINE, Scopus, Web of Science, and Cochrane library from inception up to 27 August 2022 for all relevant published articles. The search was applied using the following MESH and non-MESH terms: (“green tea” OR “green tea extract” OR “catechin” OR “catechins” OR “*Camellia sinensis”* OR “Thea sinensis”) AND (Intervention OR “Intervention Study” OR “Intervention Studies” OR “controlled trial” OR randomized OR randomized OR random OR randomly OR placebo OR “clinical trial” OR Trial OR “randomized controlled trial” OR “randomized clinical trial” OR RCT OR blinded OR “double blind” OR “double blinded” OR trial OR “clinical trial” OR trials OR “Pragmatic Clinical Trial” OR “Cross-Over Studies” OR “Cross-Over” OR “Cross-Over Study” OR parallel OR “parallel study” OR “parallel trial”) (Supplementary File 1). No restriction was considered on time and language of publications. Reference lists of the related papers were also manually checked to prevent missing any pertinent papers. In addition, duplicate citations were removed after including all searched articles in the Endnote software.

### Inclusion criteria and exclusion criteria

The inclusion criteria for the present review are listed as follows: (a) randomized clinical trials (RCT) (either parallel or cross-over design), (b) investigations on adult population (age > 18y), (c) studies that administered any types of green tea supplement, (d) clinical trials with at least one week’s of the follow-up period, and (e) articles that provided sufficient information on the baseline and final levels of cardiovascular risk factors or represented required information for calculation of those effect sizes. In the case of more than one published article for one dataset, we included the most complete one. If there were clinical studies with an extra intervention group, we considered them as two separate investigations. The following criteria were also considered to exclude studies: (a) experimental, (b) those studies with a cohort, cross-sectional, and case-control design, (c) review articles, (d) ecological investigations, (e) clinical trials with no random allocation and no control group, and (f) investigations carried out on children and adolescents.

### Data extraction

Data extraction including author’s name, publication year and the country where the study was performed, participants’ health condition, age, sex, body mass index (BMI), study design (parallel/cross-over), number of contributors in each study group, dose, and duration of prune administration, post-intervention mean and standard deviation (SD) of cardiovascular risk factors in both prune and control groups, post-intervention mean (SD) changes in cardiovascular risk factors in both study groups, and confounders adjusted in the analysis was completed by two researchers independently. If standard errors (SEs) or interquartile ranges were reported, we converted them to SDs. In addition, a chief researcher settled any controversies.

### Quality assessment

We systematically evaluated the bias in the included trials by using the Cochrane Collaboration’s tool risk of bias criteria (26). Two independent investigators assessed the quality details of the studies in seven domains including random sequence generation, allocation concealment, reporting bias, performance bias, detection bias, attrition bias, and other sources of bias based on the Cochrane Handbook for Systematic Reviews. To assess each domain, the terms “Low”, “High”, or “Unclear” was applied (Table 2).

### Statistical analysis

The overall effect sizes were computed as mean differences and SDs of glycemic markers and CRP between prune and control groups. All data were inserted as means ± SD. It should be noted that in studies where findings have been reported as SEs and interquartile ranges, means ± SD was calculated by statistical computations. The effect sizes were indicated as standardized mean difference (SMD) and 95% confidence interval (CI). The random-effects model considering between-study variations was chosen to acquire the overall effect sizes. Heterogeneity between studies was evaluated by I-square (I^2^) index and I^2^ > 50% was assumed as considerable between-study heterogeneity (27). Subgroup analysis was performed to find any probable sources of heterogeneity based on the predefined variables including duration of supplementation (≥ 12 *vs.* < 12 weeks), the dose of GTE (≥ 1,000 *vs.* < 1,000 mg/d), sex (male, female, and both), baseline TG (≥ 150 *vs.* < 150 mg/dl), TC (≥ 200 *vs.* < 200 mg/dl), LDL (≥ 100 *vs.* < 100 mg/dl), HDL (≥ 50 *vs.* < 50 mg/dl), FBS (≥ 100 *vs.* < 100 mg/dl), and HbA1c (≥ 6.5 *vs.* < 6.5%), past medical history of type 2 diabetes mellitus (T2DM; Non-T2DM patients and T2DM patients), and baseline values of BMI (normal, overweight, and obese), DBP (≥ 80 *vs.* < 80 mmHg) and SBP (≥ 130 *vs.* < 130 mmHg). Fractional polynomial modeling was applied to detect the non-linear effects of green tea dosage (g/d) on each variable level. We performed a sensitivity analysis to identify the impact of one single study removal on the overall effect size. Publication bias evaluation was carried out through visual inspection of funnel plot for each variable and statistical tests including Begg’s adjusted rank correlation and Egger’s regression asymmetry tests (28, 29). All statistical analysis was accomplished by STATA^®^ version 14.0 (StataCorp, College Station, Lakeway, TX, USA) and P-value less than 0.05 was assumed statistically significant.

### Certainty assessment

We graded the overall certainty of evidence across the studies based on the guidelines of the GRADE (Grading of Recommendations Assessment, Development, and Evaluation) Working Group. According to the corresponding evaluation criteria, four categories of high, moderate, low, and very low represented the quality of evidence.

## Results

### Study selection

**FIGURE 1:**
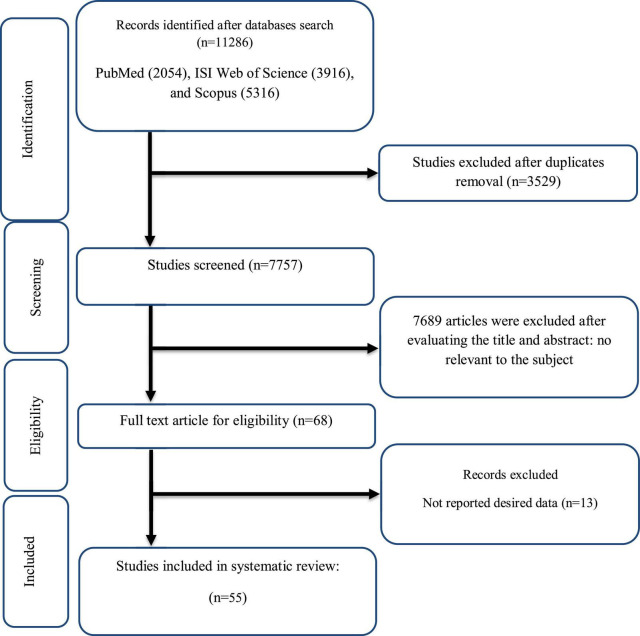
Flow chart of study selection for inclusion trials in the systematic review.

As disclosed in Figure 1, our initial search found a total of 11,286 relevant papers, of which 68 remained after duplicates removing (*n* = 3,529), and a wide range of screening of the titles and abstracts (*n* = 7,689). The entire of suitable articles were carefully checked and 13 irrelevant studies were excluded. Finally, 55 eligible clinical trials with 63 effect sizes were included in the present quantitative review based on the research topic.

### Characteristics of the included studies

Table 1 represented the characteristics of all included studies. Overall, clinical trials with a total of 4,874 participants were included (2,487 participants in the green tea group and 2,387 in the placebo group), participants’ mean age ranged between 18 and 68.7 years, and the period of intervention ranged between two to 48 weeks. Some of the studies enrolled only males or females and some of them included both genders. In addition, participants with various health conditions were enrolled in included clinical trials. Twenty-nine studies involved healthy participants (24, 30–58), 15 recruited diabetic patients (59–72), two enrolled patients with hypercholesterolemia (73, 74), two included polycystic ovarian syndrome (PCOS) patients (75, 76), two recruited obese patients with hypertension (77, 78), three recruited patients with liver disorders (79–81), one involved obese patients with metabolic syndrome (82), and another study investigated the effect of GTE supplementation on patients with chronic stable angina (83). In addition, mentioned clinical trials were executed in different countries including Australia, USA, Iran, Brazil, UK, China, Spain, Japan, Taiwan, Lithuania, Poland, Netherlands, Finland, Pakistan, and Mexico.

**TABLE 1:** Characteristics of the included studies.

Studies	Country	Study design	Participant	Sex	Sample size		Trial duration (Week)	Means age (Year)		Means BMI (kg/m^2^)		Intervention		
					IG	CG		IG	CG	IG	CG	GT type	GT dose (mg/day)	EGCG dose (mg/day)
Freese et al. (52)	Finland	DB/R/PL	Healthy females	F	10	10	4	32.8	34.3	22.3	22.8	GTE	3000	
Maron et al. (74)	China	DB/R/PL	Subjects with mild to moderate hypercholesterolemia	F/M	114	106	8	54.4	55	24	24.4	capsule containing theaflavin-enriched GTE	375	
Kovacs et al. (36)	Netherlands	RCT	Overweight and moderately obese male and female subjects	F/M	70	34	13	18-60	18-60	25-35	25-35	GTE	2700	323
Fukino et al. (59)	Japan	RCT	T2DM patients	F/M	33	33	8	53.5	53.5	25.5	25.9	mixture of GTE and green tea powder	544	
Westerterp-Plantenga et al. (51) (A)	Netherlands	DB/R/PL	Overweight and moderately obese subjects	F/M	19	19	13	18-60	18-61	29.6	29.5	GT caffeine mixture	270	270
Westerterp-Plantenga et al. (51) (B)	Netherlands	DB/R/PL	Overweight and moderately obese subjects	F/M	19	19	13	18-60	18-61	29.6	29.5	GT caffeine mixture	271	271
Chan et al. (75)	China	RCT	Obese patients with PCOS	F	17	17	12	34.8	34.8	30.9	30.9	GTE	540	540
Diepvens et al. (33)	Netherlands	DB/R/PL	Overweight female subjects	F	23	23	8	41.7	41.6	27.7	27.7	GTE	310	
Hill et al. (26)	Australia	RCT	Overweight or obese postmenopausal women	F	19	19	12	45-70	45-70	25-39.9	25-39.9	EGCG	300	300
Nagao et al. (40)	Japan	DB/R/PL	Women and men with visceral fat-type obesity	F/M	123	117	12	41.7	41.7	26.8	26.8	GTE	583	
Fukino et al. (60)	Japan	RCT/cross-over	T2DM patients	F/M	60	60	8	53.9	53.4	25.4	26	mixture of GTE and GT powder	544	
Hsu et al. (34)	Taiwan	DB/R/PL	Obese women	F	41	37	12	43	43.9	31.2	30.5	GTE	1200	
Brown et al. (30)	UK	DB/R/PL	Overweight or obese male subjects	M	46	42	8	52.15	50.57	31.21	30.96	EGCG	800	800
Nagao et al. (61)	Japan	DB/R	T2DM patients	F/M	23	20	12	64.9	62.8	NA	NA	mixture of GTE and brewed green tea	582.8	
Hursel and Westerterp-Plantenga, (50) (A)	Netherlands	DB/R/PL	Overweight and moderately obese subjects	F/M	40	40	13	44	44	29.6	29.6	GT caffeine mixture	270	270
Hursel and Westerterp-Plantenga, (50) (B)	Netherlands	DB/R/PL	Overweight and moderately obese subjects	F/M	40	40	13	44	44	29.6	29.6	GT–caffeine mixture	271	271
Frank et al. (24)	UK	RCT	Healthy men	M	17	16	3	41	40	26.7	25.4	GTE	2304	
Nantz et al. (49)	USA	DB/R/PL	Healthy adult	F/M	61	63	3	28.9	30	25.4	24.3	decaffeinated GTE	400	
Mohammadi et al. (62)	Iran	DB/R/PL	T2DM patients	F/M	29	29	8	55.14	55.14	28.64	29.37	GTE	1500	
Sone et al. (44)	Japan	RCT	Those who participated in a weight loss program at Sendai Health Promotion Center	F/M	25	26	9	43.2	48.2	24.6	24.5	catechin-enriched GT	400	
Hsu et al. (63)	Taiwan	DB/R/PL	T2DM patients	F/M	35	33	16	50.5	52.2	NA	NA	Decaffeinated GTE	1500	856.8
Brown et al. (31)	UK	DB/R/PL cross-over	Overweight and obese men	M	67	70	6	49.5	49.4	31.7	31.4	GTE	1060	800
Basu et al. (82)	USA	SB/R/PL	Obese subjects with metabolic syndrome	F/M	10	12	8	39.5	44.6	36.1	36.1	GTE	500	250
Bogdanski et al. (77)	Poland	DB/R/PL	Obese, hypertensive patients	F/M	28	28	12	42.9	51.5	32.5	33.9	GTE	379	208
Suliburska et al. (41)	Poland	DB/R/PL	Obese Patients	F/M	23	23	12	48.56	52.26	32.7	33.45	GTE	379	208
Wu et al. (43) (A)	USA	DB/R/PL	Healthy postmenopausal women	F	37	16	8	59.6	57.7	29.9	29.1	GTE	400	
Wu et al. (43) (B)	USA	DB/R/PL	Healthy postmenopausal women	F	34	16	8	62	57.7	28	29.1	GTE	800	
Miyazaki et al. (39)	Japan	RCT	Active older people	F/M	25	25	14	68.7	68.7	22.3	23	GTE	630.9	228
Fukuzawa et al. (79)	Japan	RCT	NASH patients	F/M	26	12	24	53.9	48.4	30.3	30.2	GTE	750	315.6
Lasaite et al. (64)	Lithuania	DB/R/PL	T2DM patients	F/M	17	14	36	57.2	56.8	NA	NA	GTE	400	
Mielgo-Ayuso et al. (38)	Spain	DB/R/PL	Obese women	F	43	40	12	19-49	19-49	33.7	34.3	GTE	300	300
Liu et al. (65)	Taiwan	DB/R/PL	T2DM patients	F/M	46	46	16	55.06	53.56	26.2	26.4	decaffeinated GTE	500	856.8
Mirzaei et al. (72)	Iran	DB/R/PL	T2DM patients	F/M	26	46	8	54.56	54.56	30.5	29.78	GTE	1500	
Chen et al. (32)	Taiwan	DB/R/PL	Women with central obesity	F	39	38	12	44.1	44.9	31	30	GTE	500	856.8
Dostal et al. (45)	USA	DB/R/PL	Overweight and obese postmenopausal women	F	117	120	48	60.9	60.6	28.5	27.9	GTE	1315	843
Dostal et al. (46)	USA	DB/R/PL	Overweight and obese postmenopausal women	F	61	60	48	60.7	60	27.9	27.6	GTE	1315	843
Borges et al. (66)	Brazil	DB/R/PL	T2DM patients	F/M	23	24	12	63	59	30.6	32.7	GTE	800	800
Lee et al. (83)	China	DB/R/PL	Patients with chronic stable angina	F/M	38	39	6	62.6	61.5	25.3	25.9	GTE	750	
Lu and Hsu, (37)	Taiwan	DB/R/PL	Post-adolescent women	F	33	31	4	28	30.2	20.7	21.7	decaffeinated GTE	1500	858.6
Samavat et al. (53)	USA	DB/R/PL	Postmenopausal women	F	463	473	48	60.02	59.65	25.16	25.01	GTE	1315	843
Nogueira et al. (78)	Brazil	DB/R/PL cross-over	Obese prehypertensive women	F	20	20	4	41.1	41.1	33.56	33.56	GTE	1500	
Mombaini et al. (76)	Iran	DB/R/PL cross-over	Women with PCOS	F	22	23	6	23.22	24.17	28.96	28.9	GT	500	
Kafeshani et al. (47)	Iran	DB/R/PL	Healthy adult men	M	16	16	6	20.94	21.19	22.6	22.82	GTE	450	
Tabatabaee et al. (80)	Iran	DB/R/PL	Non-alcoholic fatty liver disease	F/M	21	24	12	41	39.5	NA	NA	GT	550	
Rostamian et al. (55)	Iran	RCT	Sedentary postmenopausal women	F	14	14	2	54	54	28.8	28.8	GTE	1200	
Hussain et al. (81)	Pakistan	RCT	Non-alcoholic fatty liver disease	F/M	40	40	12	25	28	29.5	28.6	GTE	1000	
de Amorim et al. (70)	Brazil	DB/R/PL	T2DM patients	F/M	16	19	20	≥ 18 years old	≥18 years old	NA	NA	green tea extract	1120	> 97% pure EGCG
Amozadeh et al. (54)	Iran	RCT	Overweight and obese females	F	13	13	8	28.14	27.12	33.44	32.7	GTE	100	
Tadayon et al. (42)	Iran	DB/R/PL	Postmenopausal women	F	39	40	4	53.7	52.9	26.9	30.1	GTE	800	
Zandi Dareh Gharibi et al. (67)	Iran	RCT	T2DM patients	F	12	10	10	50.66	55.9	32.6	34.61	GTE	1500	
Hosseini et al. (71) (A)	Iran	DB/R/PL	T2DM patients	F/M	20	10	8	52.25	55.25	29.48	28.35	EGCG	300	300
Hosseini et al. (71) (B)	Iran	DB/R/PL	T2DM patients	F/M	20	10	8	53.6	55.25	29.59	28.35	EGCG	300	300
Maeda-Yamamoto et al. (48) (A)	Japan	DB/R/PL	Healthy adults	F/M	38	37	12	49.8	48.5	23.9	23.3	GTE	322.2	322.2
Maeda-Yamamoto et al. (48) (B)	Japan	DB/R/PL	Healthy adults	F/M	39	37	12	49.5	48.5	23.3	23.3	GTE	323.6	323.6
Huang et al. (73)	Taiwan	DB/R/PL cross-over	Overweight and obese women with high levels of LDL-C	F	36	37	6	53.1	56.8	29.1	27.9	GTE	856.8	856.8
Azizbeigi et al. (129)	Iran	RCT	Obese men	M	10	10	8	23.9	22.8	31.8	30.8	GTE	500	
Sobhani et al. (68) (A)	Iran	DB/R/PL	T2DM patients	F	11	11	8	62.52	60.82	26.82	26.88	GT	1500	
Sobhani et al. (68) (B)	Iran	DB/R/PL	T2DM patients	F	11	11	8	60.91	62.75	27.7	27.6	GT + excercise	1500	
Quezada-Fernandez et al. (69)	Mexico	DB/R/PL	T2DM patients	F/M	10	10	12	50.2	56.1	29.8	30.4	Decaffeinated GTE	400	
Bagheri et al. (57)	Iran	DB/R/PL	Overweight middle-aged men	M	15	15	8	44.6	43.8	27.3	27.2	GTE	500	45%
Bagheri et al. (58)	Iran	DB/R/PL	Overweight female	F	10	10	8	37.6	39.5	27.49	26.7	GTE	500	45%
Zhang et al. (56)	Japan	DB/R/PL	Overweight and obese men	M	12	12	12	42.5	37.2	28.4	27.7	GTE	300	300
Bazyar et al. (130)	Iran	DB/R/PL	T2DM patients	F/M	22	22	8	51.75	52.61	29.46	29.28	GTE	60	

R, randomized; PL, placebo-controlled; DB, double blind; RCT, randomized clinical trials; M, male; F, female; IG, intervention group; CG, control group; NA, not available; BMI, body mass index; GT, green tea; GTE, green tea extract, T2DM, type 2 diabetes mellitus; LDL-C, low-density cholesterol; PCOS, polycystic ovarian syndrome; NASH, non-alcoholic steatohepatitis; EGCG, epigallocatechin gallate.

**TABLE 2:** Risk of bias assessment.

Studies	Random sequence generation	Allocation concealment	Selective reporting	Other sources of bias	Blinding (participants and personnel)	Blinding (outcome assessment)	Incomplete outcome data	*General risk of bias
Freese et al. (52)	L	L	H	L	L	U	L	L
Maron et al. (74)	L	L	L	L	L	U	L	L
Kovacs et al. (36)	L	L	H	L	U	U	L	L
Fukino et al. (59)	U	U	H	H	U	U	L	U
Westerterp-Plantenga et al. (51) (A)	U	U	H	H	L	U	H	U
Westerterp-Plantenga et al. (51) (B)	U	U	H	H	L	U	H	U
Chan et al. (75)	L	U	H	L	U	U	L	L
Diepvens et al. (33)	U	U	H	L	L	U	L	U
Hill et al. (35)	U	L	H	L	U	U	L	U
Nagao et al. (40)	L	L	H	L	L	L	L	L
Fukino et al. (60)	U	U	H	L	H	H	L	U
Hsu et al. (34)	L	L	H	L	L	U	L	L
Brown et al. (30)	L	L	H	L	L	U	L	L
Nagao et al. (61)	U	L	H	L	L	U	L	U
Hursel and Westerterp-Plantenga, (50) (A)	U	L	H	L	L	U	L	U
Hursel et al. (50) (B)	U	L	H	L	L	U	L	U
Frank et al. (24)	L	L	H	H	L	U	L	L
Nantz et al. (49)	U	U	H	H	L	U	H	U
Mohammadi et al. (62)	U	L	H	L	L	U	L	U
Sone et al. (44)	L	L	H	L	L	U	L	L
Hsu et al. (63)	L	L	H	H	L	U	L	L
Brown et al. (31)	L	L	H	L	L	U	H	L
Basu et al. (82)	L	L	H	H	H	U	H	L
Bogdanski et al. (77)	L	L	H	H	L	U	L	L
Suliburska et al. (41)	L	U	H	H	L	U	H	L
Wu et al. (43) (A)	L	L	H	H	L	U	L	L
Wu et al. (43) (B)	L	L	H	H	L	U	L	L
Miyazaki et al. (39)	L	U	H	L	U	U	L	L
Fukuzawa et al. (79)	L	U	H	H	U	U	H	L
Lasaite et al. (64)	U	L	H	H	L	U	L	U
Mielgo-Ayuso et al. (38)	L	L	H	H	L	L	L	L
Liu et al. (65)	U	L	H	H	L	U	L	U
Mirzaei et al. (72)	U	U	H	H	L	U	L	U
Chen et al. (32)	L	L	H	L	L	U	L	L
Dostal et al. (45)	L	L	H	L	L	U	L	L
Dostal et al. (46)	L	L	H	L	L	U	L	L
Borges et al. (66)	L	L	H	H	L	U	H	L
Lee et al. (83)	L	L	H	L	L	U	H	L
Lu and Hsu, (37)	L	L	H	H	L	H	L	L
Samavat et al. (53)	L	L	H	L	L	L	H	L
Nogueira et al. (78)	L	U	H	H	L	U	H	L
Mombaini et al. (76)	U	U	H	L	L	U	L	U
Kafeshani et al. (47)	U	L	H	L	L	U	H	U
Tabatabaee et al. (80)	L	U	H	H	L	U	L	L
Rostamian and Bijeh, (55)	U	U	H	H	U	U	L	U
Hussain et al. (81)	L	U	H	H	H	H	H	L
de Amorim et al. (70)	L	L	H	L	L	L	L	L
Amozadeh et al. (54)	U	L	H	L	H	U	L	U
Tadayon et al. (42)	L	H	H	H	L	U	H	L
Zandi Dareh Gharibi et al. (67)	U	U	H	H	H	U	L	U
Hosseini et al. (71) (A)	L	L	H	L	L	U	L	L
Hosseini et al. (71) (B)	L	L	H	L	L	U	L	L
Maeda-Yamamoto et al. (48) (A)	L	L	H	H	L	L	H	L
Maeda-Yamamoto et al. (48) (B)	L	L	H	H	L	L	H	L
Huang et al. (73)	L	L	H	H	L	U	L	L
Azizbeigi et al. (129)	U	U	H	L	U	U	H	U
Sobhani et al. (68) (A)	L	L	H	L	L	U	L	L
Sobhani et al. (68) (B)	L	L	H	L	L	U	L	L
Quezada-Fernández et al. (69)	L	L	L	H	L	U	L	L
Bagheri et al. (57)	U	L	H	L	L	U	L	U
Bagheri et al. (58)	U	L	H	L	L	U	L	U
Zhang et al. (56)	L	L	H	H	L	U	H	L
Bazyar et al. (130)	L	U	H	L	L	U	L	L

L, low risk of bias; H, high risk of bias; U, unclear risk of bias.

*General low risk < 2 high risk, General moderate risk = 2 high risk, General high risk > 2 high risk.

### Meta-analysis results

#### Effects of green tea supplementation on TG

**FIGURE 2:**
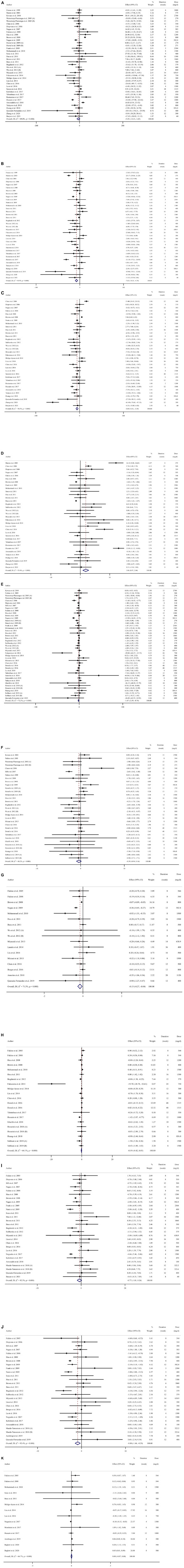
Forest plot detailing weighted mean difference and 95% confidence intervals (CIs) for the effects of green tea extract supplementation on **(A)** TG (mg/dL); **(B)** TC (mg/dL); **(C)** LDL (mg/dL); **(D)** HDL (mg/dL); **(E)** FBS (mg/dL); **(F)** fasting insulin (μlU/ml); **(G)** HbA1c (%); **(H)** HOMA-IR; **(I)** SBP (mmHg), **(J)** DBP (mmHg); **(K)** CRP (mg/dL).

green tea supplementation had a non-significant effect on TG (WMD = −5.31; 95% CI: −12.32, 1.68; *P* = 0.137) based on our analysis of 40 arms of clinical trials (Figure 2A). Moreover, remarkable heterogeneity was observed between studies (*P* = < 0.001, I^2^ = 89.0%). Subgroup analysis was carried out according to the duration and dosage of supplementation, baseline values of BMI and TG, past medical history of T2DM, and sex (Table 3). The findings of subgroup analysis suggested that green tea supplementation contributed to a significant reduction in TG if both males and females were included and the duration of intervention was more than 12 weeks. However, there was no significant effect of green tea supplementation on TG after subgroup analysis by a dose of intervention, baseline values of BMI and TG, past medical history of T2DM, and sex.

**TABLE 3:** Subgroup analyses of green tea extract supplementation on some cardiovascular risk factors in adults.

	Number of studies	WMD (95% CI)	*P*-value	Heterogeneity	
				P heterogeneity	I^2^
**Subgroup analyses of green tea extract supplementation on TG (mg/dL)**					
Overall effect	40	−5.31 (−12.32, 1.68)	0.137	<0.001	89.0%
**Baseline BMI (kg.m^–2^)**					
Normal weight (18.5-24.9)	6	−3.29 (−10.06, 3.47)	0.340	0.572	0.0%
Overweight (25-29.9)	20	−6.29 (−15.86, 3.26)	0.197	<0.001	87.0%
Obese (≥30)	11	−3.38 (−17.53, 10.76)	0.639	<0.001	88.5%
**Trial duration (week)**					
≤12	19	2.23 (−6.11, 10.57)	0.600	<0.001	81.0%
>12	21	−12.61 (−22.03, −3.19)	**0.009**	<0.001	87.4%
**Intervention dose (mg/day)**					
<1,000	29	−7.76 (−15.98, 0.44)	0.064	<0.001	86.8%
≥1,000	11	1.63 (−13.14, 16.41)	0.828	<0.001	91.8%
**Sex**					
Female	14	0.15 (−6.86, 7.18)	0.965	<0.001	71.9%
Both	21	−12.33 (−21.23, −3.43)	**0.007**	<0.001	77.3%
Male	5	5.50 (−11.93, 22.95)	0.536	<0.001	80.1%
**T2DM status**					
Non-T2DM	33	−3.92 (−11.42, 3.57)	0.305	<0.001	89.7%
T2DM patients	7	−14.83 (−31.27, 1.60)	0.077	0.059	50.5%
**Baseline TG**					
<150	26	−2.55 (−9.58, 4.47)	0.476	<0.001	86.8%
≥150	13	−12.33 (−28.07, 3.40)	0.125	<0.001	82.7%
**Subgroup analyses of green tea extract supplementation on TC (mg/dL)**					
Overall effect	36	−7.62 (−10.51, −4.73)	<0.001	<0.001	90.9%
**Baseline BMI (kg.m^–2^)**					
Normal weight (18.5-24.9)	6	−10.44 (−21.67, 0.77)	0.068	<0.001	88.3%
Overweight (25-29.9)	17	−9.27 (−14.12, −4.41)	<0.001	<0.001	83.4%
Obese (≥30)	10	−2.28 (−5.11, 0.54)	0.113	<0.001	75.1%
**Trial duration (week)**					
≤12	20	−5.80 (−9.06, −2.54)	<0.001	<0.001	88.6%
>12	16	−10.90 (−16.78, −5.02)	<0.001	<0.001	89.5%
**Intervention dose (mg/day)**					
<1000	26	−7.48 (−10.77, −4.19)	<0.001	<0.001	91.4%
≥1000	10	−7.43 (−14.90, 0.04)	0.051	<0.001	87.0%
**Sex**					
Female	14	−4.69 (−8.60, −0.78)	0.019	<0.001	64.5%
Both	17	−11.17 (−16.61, −5.74)	<0.001	<0.001	95.2%
Male	5	−1.39 (−4.53, 1.75)	0.387	0.328	13.6%
**T2DM status**					
Non-T2DM	29	−7.20 (−10.22, −4.18)	<0.001	<0.001	90.2%
T2DM patients	7	−9.90 (−18.70, −1.10)	0.027	<0.001	82.8%
**Baseline TC (mg/dL)**					
<200	14	−2.64 (−5.36, 0.07)	0.057	0.010	53.2%
≥200	22	−10.64 (−15.53, −5.75)	<0.001	<0.001	92.6%
**Subgroup analyses of green tea extract supplementation on LDL (mg/dL)**					
Overall effect	34	−5.80 (−8.30, −3.30)	<0.001	<0.001	90.5%
**Baseline BMI (kg.m^–2^)**					
Normal weight (18.5-24.9)	4	−3.49 (−7.79, 0.80)	0.111	0.458	0.0%
Overweight (25-29.9)	17	−8.40 (−12.45, −4.34)	<0.001	<0.001	84.5%
Obese (≥30)	11	−2.91 (−6.84, 1.00)	0.145	<0.001	93.2%
**Trial duration (week)**					
≤12	18	−3.59 (−6.08, −1.10)	0.005	<0.001	84.5%
>12	16	−10.05 (−16.61, −3.49)	0.003	<0.001	92.4%
**Intervention dose (mg/day)**					
<1000	26	−5.21 (−8.08, −2.34)	<0.001	<0.001	90.2%
≥1000	8	−7.41 (−11.74, −3.08)	0.001	<0.001	75.3%
**Sex**					
Female	13	−4.22 (−9.41, 0.97)	0.112	<0.001	86.3%
Both	17	−5.99 (−9.51, −2.47)	0.001	<0.001	84.6%
Male	4	−16.25 (−34.21, 1.70)	0.076	<0.001	97.0%
**T2DM status**					
Non-T2DM	28	−6.32 (−9.09, −3.56)	<0.001	<0.001	92.0%
T2DM patients	6	−2.66 (−7.78, 2.44)	0.307	0.146	39.0%
**Baseline LDL (mg/dL)**					
<100	3	−5.38 (−7.70, −3.07)	<0.001	0.757	0.0%
≥100	30	−6.01 (−8.80, −3.21)	<0.001	<0.001	91.3%
**Subgroup analyses of green tea extract supplementation on HDL (mg/dL)**					
Overall effect	34	1.85 (0.87, 2.84)	0.010	<0.001	94.4%
**Baseline BMI (kg.m^–2^)**					
Normal weight (18.5-24.9)	5	3.70 (−1.81, 9.22)	0.188	<0.001	91.2%
Overweight (25-29.9)	16	1.07 (−1.44, 3.59)	0.405	<0.001	92.2%
Obese (≥30)	11	1.66 (0.53, 2.78)	0.004	<0.001	93.1%
**Trial duration (week)**					
≤12	18	0.73 (−0.02, 1.68)	0.126	<0.001	87.7%
>12	16	2.96 (0.61, 5.30)	0.013	<0.001	93.0%
**Intervention dose (mg/day)**					
<1000	25	1.72 (0.73, 2.70)	0.001	<0.001	91.3%
≥1000	9	1.82 (−1.98, 4.62)	0.348	<0.001	95.3%
**Sex**					
Female	13	1.63 (0.15, 3.11)	0.030	<0.001	80.1%
Both	16	2.79 (−0.09, 5.67)	0.058	<0.001	95.9%
Male	5	−0.48 (−2.37, 1.39)	0.611	0.012	68.7%
**T2DM status**					
Non-T2DM	28	2.16 (1.07, 3.26)	<0.001	<0.001	94.5%
T2DM patients	6	0.11 (−1.24, 1.48)	0.866	0.486	0.0%
**Baseline HDL (mg/dL)**					
>50	18	2.40 (1.14, 3.67)	<0.001	<0.001	95.3%
≥50	15	1.30 (−0.89, 3.49)	0.246	<0.001	87.8%
**Subgroup analyses of green tea extract supplementation on FBS (mg/dL)**					
Overall effect	44	−1.67 (−2.58, −0.75)	<0.001	<0.001	72.2%
**Baseline BMI (kg.m^–2^)**					
Normal weight (18.5-24.9)	3	0.37 (−3.54, 4.29)	0.851	0.074	61.6%
Overweight (25-29.9)	22	−1.61 (−2.95, −0.28)	0.018	0.099	29.2%
Obese (≥30)	15	−0.90 (−1.85, 0.04)	0.060	<0.001	69.5%
**Trial duration (week)**					
≤12	21	0.03 (−0.26, 0.33)	0.831	0.510	0.0%
>12	23	−2.64 (−4.39, −0.89)	0.003	<0.001	78.9%
**Intervention dose (mg/day)**					
<1000	30	−1.80 (−2.88, −0.72)	0.001	<0.001	79.8%
≥1000	14	−0.83 (−2.07, 0.41)	0.193	0.629	0.0%
**Sex**					
Female	17	−1.52 (−2.91, −0.13)	0.031	0.006	52.7%
Both	24	−2.10 (−4.20, −0.01)	0.049	<0.001	78.3%
Male	3	0.08 (−0.26, 0.44)	0.630	0.670	0.0%
**T2DM status**					
Non-T2DM	30	−1.13 (−1.90, −0.37)	0.004	<0.001	63.8%
T2DM patients	14	−2.72 (−9.05, 3.60)	0.399	0.002	59.9%
**Baseline FBS (mg/dL)**					
<100	19	−1.22 (−2.09, −0.35)	0.006	<0.001	71.3%
≥100	24	−2.26 (−5.00, 0.47)	0.106	<0.001	69.2%
**Subgroup analyses of green tea extract supplementation on fasting insulin (μlU/ml)**					
Overall effect	32	−0.39 (−0.94, 0.16)	0.165	<0.001	68.2%
**Baseline BMI (kg.m^–2^)**					
Overweight (25-29.9)	17	−0.27 (−0.80, 0.24)	0.300	0.038	41.5%
Obese (≥30)	11	−0.37 (−1.52, 0.77)	0.521	<0.001	80.5%
**Trial duration (week)**					
≤12	14	−0.45 (−1.09, 0.19)	0.170	0.276	16.3%
>12	18	−0.30 (−1.07, 0.47)	0.447	<0.001	79.2%
**Intervention dose (mg/day)**					
<1000	20	−0.47 (−1.33, 0.38)	0.278	<0.001	77.4%
≥1000	12	−0.01 (−0.39, 0.36)	0.947	0.632	0.0%
**Sex**					
Female	13	−0.78 (−1.71, 0.14)	0.099	<0.001	78.2%
Both	17	−0.04 (−0.74, 0.66)	0.911	0.015	47.9%
Male	2	−0.04 (−0.96, 0.87)	0.923	0.754	0.0%
**T2DM status**					
Non-T2DM	19	−0.54 (−1.22, 0.12)	0.111	<0.001	77.5%
T2DM patients	13	0.08 (−0.78, 0.95)	0.843	0.224	21.7%
**Subgroup analyses of green tea extract supplementation on HbA1c (%)**					
Overall effect	17	−0.15 (−0.26, −0.04)	0.008	<0.001	71.3%
**Baseline BMI (kg.m^–2^)**					
Normal weight (18.5-24.9)	1	−0.20 (−0.65, 0.25)	0.391	–	–
Overweight (25-29.9)	7	−0.26 (−0.58, 0.06)	0.117	0.126	39.9%
Obese (≥30)	5	−0.06 (−0.09, −0.04)	<0.001	0.433	0.0%
**Trial duration (week)**					
≤12	8	−0.14 (−0.28, −0.00)	0.049	0.109	40.5%
>12	9	−0.13 (−0.32, 0.05)	0.154	0.001	69.6%
**Intervention dose (mg/day)**					
<1000	13	−0.10 (−0.22, 0.00)	0.063	<0.001	73.4%
≥1000	4	−0.51 (−0.82, −0.19)	0.001	0.370	4.6%
**Sex**					
Female	3	−0.10 (−0.33, 0.13)	0.394	1.000	0.0%
Both	13	−0.19 (−0.35, −0.02)	0.021	<0.001	68.5%
Male	1	−0.07 (−0.09, −0.04)	<0.001	–	–
**T2DM status**					
Non-T2DM	6	−0.06 (−0.09, −0.04)	<0.001	0.962	0.0%
T2DM patients	11	−0.22 (−0.41, −0.03)	0.019	0.001	68.0%
**Baseline HbA1c (%)**					
<6.5	8	−0.07 (−0.09, −0.04)	<0.001	0.974	0.0%
≥6.5	8	−0.23 (−0.47, 0.01)	0.061	<0.001	77.4%
**Subgroup analyses of green tea extract supplementation on HOMA-IR**					
Overall effect	21	−0.18 (−0.42, 0.05)	0.122	<0.001	64.1%
**Baseline BMI (kg.m^–2^)**					
Overweight (25-29.9)	12	−0.14 (−0.43, 0.14)	0.320	0.002	
Obese (≥30)	7	−0.32 (−0.87, 0.22)	0.249	62.5%	76.7%
**Trial duration (week)**					
≤12	10	0.08 (−0.16, 0.32)	0.519	0.406	3.7%
>12	11	−0.35 (−0.68, −0.01)	0.040	<0.001	77.8%
**Intervention dose (mg/day)**					
<1000	12	−0.15 (−0.57, 0.26)	0.472	0.001	64.0%
≥1000	9	−0.23 (−0.55, 0.09)	0.161	0.002	68.1%
**Sex**					
Female	9	−0.01 (−0.12, 0.08)	0.769	0.638	0.0%
Both	11	−0.33 (−1.04, 0.37)	0.358	<0.001	78.5%
**T2DM status**					
Male	1	0.00 (−0.30, 0.30)	1.000	–	–
Non-T2DM	11	−0.29 (−0.59, 0.00)	0.053	<0.001	77.8%
T2DM patients	10	0.11 (−0.22, 0.45)	0.514	0.399	4.5%
**Subgroup analyses of green tea extract supplementation on SBP (mmHg)**					
Overall effect	28	−0.77 (−1.80, 0.26)	0.144	<0.001	92.3%
**Baseline BMI (kg.m^–2^)**					
Normal weight (18.5-24.9)	6	0.73 (−1.82, 3.30)	0.571	0.095	46.7%
Overweight (25-29.9)	9	−0.91 (−3.14, 1.30)	0.417	0.001	68.6%
Obese (≥30)	11	−1.30 (−2.78, 0.17)	0.083	<0.001	96.4%
**Trial duration (week)**					
≤12	14	−1.28 (−2.61, 0.04)	0.059	<0.001	79.8%
>12	14	−0.59 (−1.69, 0.50)	0.287	<0.001	67.8%
**Intervention dose (mg/day)**					
<1000	22	−0.94 (−1.98, 0.09)	0.075	<0.001	90.2%
≥1000	6	0.31 (−3.41, 4.04)	0.868	<0.001	86.4%
**Sex**					
Female	7	−0.70 (−3.18, 1.78)	0.580	<0.001	88.4%
Both	17	−0.42 (−1.86, 1.01)	0.563	<0.001	81.7%
Male	4	−2.02 (−3.40, −0.64)	0.004	0.244	28.0%
**T2DM status**					
Non-T2DM	20	−1.38 (−2.45, −0.32)	0.011	<0.001	79.5%
T2DM patients	8	0.36 (−1.13, 1.86)	0.633	0.069	46.7%
**Baseline SBP (mmHg)**					
<130	12	0.12 (−1.17, 1.42)	0.855	0.059	42.4%
≥130	14	−1.42 (−2.86, 0.00)	0.051	<0.001	95.8%
**Subgroup analyses of green tea extract supplementation on DBP (mmHg)**					
Overall effect	28	−0.87 (−1.45, −0.29)	0.003	<0.001	92.4%
**Baseline BMI (kg.m^–2^)**					
Normal weight (18.5-24.9)	6	−0.32 (−1.87, 1.22)	0.680	0.759	0.0%
Overweight (25-29.9)	8	−0.49 (−2.41, 1.43)	0.618	<0.001	79.1%
Obese (≥30)	12	−0.88 (−1.61, −0.15)	0.018	<0.001	96.2%
**Trial duration (week)**					
≤12	14	−1.16 (−2.28, −0.04)	0.042	<0.001	95.2%
>12	14	−0.45 (−1.28, 0.38)	0.287	<0.001	69.7%
**Intervention dose (mg/day)**					
<1,000	21	−0.99 (−1.90, −0.09)	0.031	<0.001	93.8%
≥1,000	6	−0.02 (−1.68, 1.63)	0.977	0.015	64.6%
**Sex**					
Female	7	−0.62 (−1.54, 0.29)	0.183	0.095	44.4%
Both	16	−1.03 (−2.13, 0.07)	0.067	<0.001	83.9%
**T2DM status**					
Male	5	−0.40 (−2.25, 1.44)	0.667	<0.001	98.2%
Non-T2DM	21	−0.74 (−1.62, 0.12)	0.094	<0.001	92.9%
T2DM patients	7	−0.72 (−2.31, 0.85)	0.368	<0.001	80.1%
**Baseline TG**					
<80	12	−0.28 (−0.97, 0.39)	0.410	0.011	54.8%
≥80	15	−1.60 (−2.43, −0.78)	<0.001	<0.001	66.7%
**Subgroup analyses of green tea extract supplementation on CRP (mg/dL).**					
Overall effect	16	−0.03 (−0.14, 0.08)	0.619	<0.001	90.2%
**Baseline BMI (kg.m^–2^)**					
Normal weight (18.5-24.9)	1	−1.09 (−3.27, 1.09)	0.329	–	–
Overweight (25-29.9)	9	0.05 (−0.42, 0.54)	0.816	<0.001	93.5%
Obese (≥30)	5	0.13 (−0.05, 0.33)	0.155	0.002	75.9%
**Trial duration (week)**					
≤12	11	−0.00 (−0.05, 0.05)	0.953	0.018	53.3%
>12	5	−0.03 (−0.83, 0.75)	0.928	<0.001	96.8%
**Intervention dose (mg/day)**					
<1000	13	0.08 (−0.02, 0.18)	0.113	<0.001	68.8%
≥1000	3	−0.24 (−1.06, 0.58)	0.565	<0.001	98.2%
**Sex**					
Female	4	0.00 (−0.07, 0.08)	0.928	0.001	81.1%
Both	10	0.05 (−0.49, 0.60)	0.851	<0.001	93.1%
Male	2	−0.24 (−0.89, 0.41)	0.468	0.073	68.8%

CI, confidence interval; WMD, weighted mean differences; BMI, body mass index; FBS, fasting blood sugar; HbA1c, hemoglobin A1C; HOMA-IR, homeostatic model assessment for insulin resistance; SBP, systolic blood pressure; DBP, diastolic blood pressure; TC, total cholesterol; TG, triglyceride; LDL-C, low-density cholesterol; HDL-C, high-density cholesterol; and CRP, C-reactive protein.

#### Effects of green tea supplementation on TC

The effect of green tea supplementation on TC was examined in 36 arms of clinical trials. Pooled mean difference from the inverse variance method demonstrated a significant decrease in TC (WMD = −7.62; 95% CI: −10.51, −4.73; *P* = < 0.001) (Figure 2B). In addition, considerable between-study heterogeneity was disclosed (*P* = < 0.001, *I*^2^ = 90.9%). Subgroup analysis was accomplished based on duration and dosage of supplementation, baseline values of BMI and TC, past medical history of T2DM, and sex (Table 3). According to the results of subgroup analysis, green tea supplementation significantly decreased TC when females or both males and females were included, the dosage of supplementation was less than 1,000 mg/d, the baseline BMI was between 25-29.9 kg.m^–2^, and the baseline value of TC was more than 200 mg/dl.

#### Effects of green tea supplementation on LDL

The overall finding of our meta-analysis on 34 arms of clinical trials demonstrated that green tea supplementation had a significant decreasing effect on LDL (WMD = −5.80; 95% CI: −8.30, −3.30; *P* = < 0.001) (Figure 2C). In addition, considerable between-study heterogeneity was found (*P* < 0.001, I^2^ = 90.5%). Subgroup analysis was performed according to the duration and dosage of supplementation, baseline values of BMI and LDL, past medical history of T2DM, and sex (Table 3). The findings of subgroup analysis suggested that green tea supplementation contributed to a significant reduction in LDL if males or both males and females were included, the baseline BMI was between 25-29.9 kg.m^–2^ and participants were not affected by T2DM.

#### Effects of green tea supplementation on HDL

The overall finding of our meta-analysis on 34 arms of clinical trials exhibited that green tea supplementation significantly increased HDL (WMD = 1.85; 95% CI: 0.87, 2.84; *P* = 0.010) (Figure 2D). Also, there was heterogeneity among studies (*P* = < 0.001, I^2^ = 94.4%). Subgroup analysis was performed according to the duration and dosage of supplementation, baseline values of BMI and HDL, past medical history of T2DM, and sex (Table 3). The results of subgroup analysis revealed a significant elevation in HDL if females were included, the baseline BMI was lower more than 30 kg.m^–2^, there was no past medical history of T2DM, the duration of intervention was more than 12 weeks, the dosage of supplementation was less than 1000 mg/d, and baseline values of HDL were more than 50 mg/dl.

#### Effects of green tea supplementation on FBS

Combining effect sizes from 44 arms of clinical trials significantly decreased FBS after green tea supplementation (WMD = −1.67; 95% CI: −2.58, −0.75; *P* = < 0.001) (Figure 2E). In addition, considerable heterogeneity was found among studies (*P* = < 0.001, I^2^ = 72.2%). Subgroup analysis was conducted based on the duration and dosage of supplementation, baseline values of BMI and FBS, past medical history of T2DM, and sex (Table 3). The findings of subgroup analysis indicated a significant decrease in FBS when the baseline BMI of participants was between 25-29.9 kg.m^–2^, female or both male and female were included, the duration of intervention was more than 12 weeks, the dosage of supplementation was less than 1000 mg/d, and baseline values of FBS were less than 100 mg/dl.

#### Effects of green tea supplementation on HbA1c

Our preliminary analysis on 17 arms of clinical trials proposed a significant decrease in HbA1c following green tea supplementation (WMD = −0.15; 95% CI: −0.26, −0.04; *P* = 0.008) (Figure 2G). Also, there was heterogeneity among included studies (P = < 0.001, I^2^ = 71.3%). Subgroup analysis was carried out based on the duration and dosage of intervention, baseline values of BMI and HbA1c, past medical history of T2DM, and sex (Table 3). A significant decrease in HbA1c was found if the duration of intervention was ≤ 12 weeks, the dosage of supplementation was ≥ 1,000 mg/d, baseline values of HbA1c were less than 6.5%, male or both genders were involved, and the baseline value of BMI was ≥ 30 kg.m^–2^.

#### Effects of green tea supplementation on fasting insulin

Non-significant effect on fasting insulin was observed following green tea supplementation (WMD = −0.39; 95% CI: −0.94, 0.16; *P* = 0.165) according to our analysis of 32 arms of clinical trials (Figure 2F). Also, there was heterogeneity among studies (*P* = < 0.001, I^2^ = 68.2%). Subgroup analysis was done based on the duration and dosage of supplementation, baseline values of BMI, past medical history of T2DM, and sex (Table 3). The results of the subgroup analysis indicated that green tea supplementation had non-significant effects on fasting insulin after subgroup analysis by all aforementioned factors.

#### Effects of green tea supplementation on HOMA-IR

Non-significant effect on HOMA-IR was observed following green tea supplementation (WMD = −0.18; 95% CI: −0.42, 0.05; *P* = 0.122) according to our analysis of 21 arms of clinical trials (Figure 2H). Also, there was heterogeneity among studies (*P* = < 0.001, I^2^ = 64.1%). Subgroup analysis was carried out based on the duration and dosage of supplementation, baseline values of BMI, past medical history of T2DM, and sex (Table 3). The results of the subgroup analysis suggested that green tea supplementation had non-significant effects on HOMA-IR after subgroup analysis by all aforementioned factors.

#### Effects of green tea supplementation on SBP

The overall finding of our meta-analysis on 28 arms of clinical trials demonstrated that green tea supplementation had no significant effect on SBP (WMD = −0.77; 95% CI: −1.80, 0.26; *P* = 0.144) (Figure 2I). In addition, considerable heterogeneity was found among studies (*P* = < 0.001, *I*^2^ = 92.3%). Subgroup analysis was accomplished based on duration and dosage of supplementation, baseline values of BMI and SBP, past medical history of T2DM, and sex (Table 3). The results of subgroup analysis reported a significant decreasing effect of green tea supplementation on SBP if the male was only included and participants were not affected by T2DM. However, there was no significant effect of green tea on SBP after subgroup analysis by the dosage and duration of intervention and baseline values of BMI and SBP.

#### Effects of green tea supplementation on DBP

A significant decreasing effect on DBP was observed following green tea supplementation (WMD = −0.87; 95% CI: −1.45, −0.29; *P* = 0.003) according to our analysis of 28 arms of clinical trials (Figure 2J). Also, remarkable heterogeneity was observed between studies (*P* < 0.001, I^2^ = 92.4%). Subgroup analysis was performed based on duration and dosage of supplementation, baseline values of BMI and DBP, past medical history of T2DM, and sex (Table 3). A significant decrease in DBP was observed if the duration of intervention was ≤ 12 weeks, the dosage of supplementation was less than 1,000 mg/d, baseline values of DBP were more than 80 mmHg, and the baseline value of BMI was ≥ 30 kg.m^–2^.

#### Effects of green tea supplementation on CRP

Non-significant effect on CRP was found following green tea supplementation (WMD = −0.03; 95% CI: −0.14, 0.08; *P* = 0.619) according to our analysis of 16 arms of clinical trials (Figure 2K). Also, there was heterogeneity among included clinical trials (*P* = < 0.001, I^2^ = 90.2%). Subgroup analysis was done based on the duration and dosage of intervention, baseline values of BMI, and sex (Table 3). The results of the subgroup analysis disclosed non-significant effects of green tea supplementation on CRP after subgroup analysis by all aforementioned factors.

#### Publication bias

Visual inspection of the funnel plot (Supplementary Figure 1) and the results of Egger’s test did not find any publication bias in clinical trials investigating the effects of green tea supplementation on TG (Egger’s test, *P* = 0.131), fasting insulin (Egger’s test, *P* = 0.645), HbA1c (Egger’s test, *P* = 0.223), HOMA-IR (Egger’s test, *P* = 0.057), SBP (Egger’s test, *P* = 0.086), DBP (Egger’s test, *P* = 0.238), and CRP (Egger’s test, *P* = 0.902). However, there was publication bias for TC (Egger’s test, *P* = 0.021), LDL (Egger’s test, *P* = 0.024), HDL (Egger’s test, *P* = 0.001), and FBS (Egger’s test, *P* = 0.019).

#### Linear and non-linear dose responses between dose and duration of green tea supplementation and cardiovascular risk factors

To assess the potential association between alterations in TG, TC, LDL, HDL, FBS, fasting insulin, HbA1c, HOMA-IR, SBP, DBP, and CRP and dose and duration of green tea supplementation, meta-regression analysis using the random-effects model was applied (Supplementary Figures 2, 3, 4, 5). Based on the findings of meta-regression analysis, there was no linear association between absolute alterations in TC, LDL, HDL, FBS, fasting insulin, HbA1c, SBP, DBP, and CRP, and dose of intervention. However there is a significant linear relationship between absolute alterations in TG and dose (Coefficient: 7.60, *P*-value = 0.049).

Also, non-linear association between absolute changes in TC, LDL, HDL, FBS, fasting insulin, HbA1c, SBP and CRP, and duration of intervention was observed. However, there was a linear association between absolute changes in TG (Coefficient: −9.43, *P*-value = 0.017), HOMA-IR (Coefficient: −2.74, *P*-value < 0.001) and DBP (Coefficient: 0.90, *P*-value = 0.037) and the duration of the intervention. In addition, a linear association between absolute changes in TG (Coefficient: 15.88, *P*-value = 0.042) and HDL (Coefficient: −3.21, *P*-value = 0.044) and the dose of intervention was found.

#### Grading of evidence

To assess the certainty of the evidence, the GRADE protocol was applied (Table 4) and obtained findings revealed that TC, LDL, HDL, FBS, HbA1c, and DBP-related evidence had moderate quality due to the serious inconsistency reasons. Additionally, it was shown that evidence regarding TG, fasting insulin, SBP, and CRP had low quality due to serious imprecision and inconsistency reasons. The evidence relating to HOMA-IR was also downgraded to very low quality because of the serious inconsistency, imprecision, and publication bias.

**TABLE 4:** GRADE profile of green tea extract supplementation for some cardiovascular risk factors in adults.

Outcomes	Risk of bias	Inconsistency	Indirectness	Imprecision	Publication bias	Number of intervention/control	Quality of evidence
TG	No serious limitation	Serious limitation^1^	No serious limitation	Serious limitation^2^	No serious limitation	3,548 (1,825/1,723)	⊕⊕○○ Low
TC	No serious limitation	Serious limitation^1^	No serious limitation	No serious limitation	No serious limitation	3,332 (1,698/1,634)	⊕⊕⊕○ Moderate
LDL-C	No serious limitation	Serious limitation^1^	No serious limitation	No serious limitation	No serious limitation	3,139 (1,595/1,544)	⊕⊕⊕○ Moderate
HDL-C	No serious limitation	Serious limitation^1^	No serious limitation	No serious limitation	No serious limitation	3,191 (1,627/1,564)	⊕⊕⊕○ Moderate
FBS	No serious limitation	Serious limitation^1^	No serious limitation	No serious limitation	No serious limitation	2,905 (1,503/1,402)	⊕⊕⊕○ Moderate
Fasting insulin	No serious limitation	Serious limitation^1^	No serious limitation	Serious limitation^2^	No serious limitation	2,190 (1,136/1,054)	⊕⊕○○ Low
HbA1c	No serious limitation	Serious limitation^1^	No serious limitation	No serious limitation	No serious limitation	992 (509/483)	⊕⊕⊕○ Moderate
HOMA-IR	No serious limitation	Serious limitation^1^	No serious limitation	Serious limitation^2^	Serious limitation^3^	1,506 (775/731)	⊕○○○ Very Low
SBP	No serious limitation	Serious limitation^1^	No serious limitation	Serious limitation^2^	No serious limitation	1,899 (958/941)	⊕⊕○○ Low
DBP	No serious limitation	Serious limitation^1^	No serious limitation	No serious limitation	No serious limitation	1,875 (946/929)	⊕⊕⊕○ Moderate
CRP	No serious limitation	Serious limitation^1^	No serious limitation	Serious limitation^2^	No serious limitation	907 (452/455)	⊕⊕○○ Low

FBS, fasting blood sugar; HbA1c, hemoglobin A1C; HOMA-IR, homeostatic model assessment for insulin resistance; SBP, systolic blood pressure; DBP, diastolic blood pressure; TC, total cholesterol; TG, triglyceride; LDL-C, low-density cholesterol; HDL-C, high-density cholesterol; and CRP, C-reactive protein.

^1^There is significant heterogeneity.

^2^There is no evidence of significant effects of green tea extract supplementation.

^3^There is significant publication bias.

#### Sensitivity analysis

Based on the sensitivity analysis findings, for all considered cardiovascular risk factors including lipid profiles, glycemic indices, SBP and DBP, and CRP, there was no significant difference in results with removing one single study.

## Discussion

In the present meta-analysis, we weighed the effects of green tea supplementation on cardiovascular risk factors, including lipid (TG, TC, HDL, and LDL) and glycemic profiles (FBS, fasting insulin, HbA1c, and HOMA-IR), BP (SBP and DBP), and CRP as the marker of systemic inflammation. According to the findings, green tea supplementation was associated with small but significant improvements in the lipid profile by decreasing TC and LDL. Interestingly, green tea supplementation resulted in increases in HDL. In terms of TG, subgroup analyses showed that green tea supplementation had significantly favorable effects on TG in long-term interventions. green tea also showed favorable effects on the glycemic profile by decreasing FBS and HbA1c without any changes in fasting insulin and HOMA-IR. Moreover, our results demonstrated a small decline in DBP, highlighting the possible hypotensive effects of green tea supplementation. However, green tea had no significant effects on CRP.

Primary observations from *in vitro* and animal studies indicate that green tea supplementation inhibits CVD processes, which suggested the possible protective role of green tea against this disease (16). Moreover, previous epidemiological studies showed the significance of drinking green tea in the prevention of CVD (84, 85). For example, Kuriyama et al. reported that green tea consumption is associated with reduced mortality due to CVD in a population-based, prospective cohort study initiated among 40,530 Japanese adults aged 40 to 79 years (10). Furthermore, in another cohort of 165,000 adult men, Liu et al. showed that regular green tea consumption is associated with a significantly reduced risk of death from all-cause, and CVD among Chinese adults (86).

Our finding on the possible favorable effects of green tea supplementation on lipid profile is similar to the previous meta-analysis. A meta-analysis by Onakpoya et al. revealed a significant reduction in TC and LDL without any changes in HDL and TG (87). These results were repeated in the more recent systematic review and meta-analysis studies (88–90). However, our findings underlined that green tea also can have positive effects on lipid profile by increasing HDL which was not seen in the previous meta-analyses. Moreover, we showed that green tea supplementation can decrease TG if intervention lasts more than 12 weeks. The possible mechanisms underlying the positive effects of green tea on lipid profile. The hypolipidemic effects of GTE can be attributed to the high content of flavonoids, especially catechins, which are potent antioxidants (91). One of these catechins high in green tea is epigallocatechin (57). It is well-known that dietary supplements with antioxidant properties may have hypolipidemic effects (92–95). In terms of green tea antioxidants, previous *in vitro* studies showed that epigallocatechin can inhibit lipoprotein oxidation, namely, against LDL oxidation (96). The GTE can improve lipid profile by reducing micellar solubility and intestinal absorption of cholesterol, and reducing hepatic cholesterol concentration (97, 98). It should be noted that another possible mechanism involved in the favorable effects of green tea consumption on lipid profile is its anti-obesity property. Previous studies indicated that weight reduction can improve lipid profiles (99, 100). Previous studies reported anti-obesity effects of green tea by a small but significant effect of green tea on body mass (101).

Large population cohort studies reported that regular green tea intake may decrease the risk of T2DM. For example, a cohort study of 0.5 million adults aged 30–79 years suggested that daily green tea consumption was associated with a lower risk of incident T2DM and a lower risk of all-cause mortality in patients with diabetes (102). A more recent prospective cohort study among the 27 841 rural community residents in Deqing County revealed that drinking green tea may reduce the risk of T2DM among the adult population in rural China (103). Regarding the hypoglycemic of green tea and its effects on glycemic profile, although most systematic reviews and meta-analyses underlined the favorable effects of green tea, there are some inconsistencies between them. For example, Xu et al. reported that GTE supplementation significantly reduced FBS without any changes in other glycemic indices (104). These findings were repeated in a more recent meta-analysis (89). In contrast, the results of our previous systematic review and meta-analysis indicated that the green tea supplementation had no significant effect on FBS, fasting insulin, HbA1c, and HOMA-IR in patients with T2DM (105). However, our findings revealed that green tea supplementation has favorable effects on the glycemic profile by decreasing both FBS and HbA1c. Although the antioxidant content and anti-obesity of green tea (which is discussed above) are involved in the favorable effects of GTE on glycemic profile, (99, 106–108) in our study, some other possible mechanisms can contribute. It has been shown that green tea can increase circulating adiponectin (91). It is well-documented that adiponectin is the most abundant peptide secreted by adipocytes, whose increases are considered a therapeutic target in obesity-related diseases, including insulin resistance and T2DM (109, 110). Therefore, the adiponectin-increasing effects of green tea can be a possible mechanism for its hypoglycemic effects.

Hypertension is one of the chief risk factors for CVD (111). There are meta-analyses studies conducted to evaluate the effects of green tea consumption on BP. Increasingly, these studies reported inconsistent findings. For example, a primary meta-analysis published in 2014 showed that GTE supplementation resulted in significant reductions in SBP but not DBP (87). Xu et al. showed that even short-term GTE supplementation significantly reduced SBP and DBP (112) which is consistent with Igho-Osagie et al. study which revealed that short-term tea and green tea consumption is not associated with a reduction in blood pressure (113). In another systematic review and meta-analysis, Mahdavi-Roshan et al. suggested the positive effects of regular green tea consumption on BP in participants with elevated BP or hypertension by decreasing both SBP and DBP (114). Recently, an umbrella review and meta meta-analysis study showed that regular consumption of green tea significantly decreases SBP and DBP (89). However, our analysis demonstrated a small decline in DBP without any changes in SBP. It should be noted that the hypotensive effects of green tea were small and may not reach clinical importance. The small hypotensive effects of green tea may be the cause of its antioxidant contents. Previous studies underlined the significant role of antioxidant agents as a hypotensive treatment (115–117). Furthermore, green tea catechin cleanses reactive oxygen and nitrogen species; it also enhances antioxidant enzymes such as catalase and superoxide dismutase, thereby protecting endothelial cells from oxidative damage and regulating BP (118, 119). Moreover, green tea has been shown to increase circulating adiponectin. Evidence suggests that adiponectin has a potent role in regulating blood pressure. Adiponectin reduces blood presure through anti-atherogenic and insulin sensitivity effects and reversed salt-induced hypertenstion (120).

A large volume of clinical data indicates that the detection of CRP is of predictive value in CVD (121, 122). It has been hypothesized that green tea has CRP-lowering effects through inhibition of the Nuclear factor kappa-light-chain-enhancer of activated B cells (NF-kB) pathway and stimulation of nitric oxide (NO) production (123–125). This hypothesis underlined in some previous studies. For example, in our previous systematic review and meta-analysis study, we indicated that GTE supplementation significantly reduced CRP in patients with T2DM (126). However, these favorable effects were not seen in other systematic reviews and meta-analyses (127, 128). Our finding also underlined that green tea had no significant effects on CRP.

This meta-analysis contains some strengths and limitations. The main strength of this study is the relatively acceptable number of studies and high sample size. Moreover, we analyzed a wide range of biomarkers that are linked to CVD. Another advantage is performing a dose-response meta-regression analysis to evaluate the association between pooled effect size, dosage, and duration of green tea supplementation. Another strength of this meta-analysis relates to the inclusion of several long-term studies, which certainly has the advantage of documenting the long-term effects of GTE on CVD markers and allowing comparisons to shorter-duration designs (e.g., TG was shown to decrease to a greater extent in studies of longer duration). Finally, we graded the overall certainty of evidence across the studies according to the GRADE guidelines. Regarding limitations, statistical heterogeneity is apparent in our analysis. This may be attributed to methodological diversity (different study designs) and/or differences in treatment regimens (doses/durations) or the intervention type (different types of green tea which is mentioned in Table 1). In addition, there was publication bias for some biomarkers is another limitation of our study.

## Conclusion

green tea supplementation was associated with a small but significant improvement in the lipid profile by decreasing TC and LDL while increasing HDL. Moreover, green tea supplementation had significantly favorable effects on TG in long-term interventions. green tea also showed favorable effects on the glycemic profile by decreasing FBS and HbA1c without any changes in fasting insulin and HOMA-IR. Moreover, our results demonstrated a small but significant decline in DBP. Moreover, green tea had no significant effects on CRP.

## Author contributions

OA contributed to the conception and design of the study, data analysis, and supervised the study. DA-L and MK contributed to the data extraction. MZ and MK screened article for inclusion criteria. MG and DA-L contributed to the manuscript drafting. RB, KG, NA, and IT revised the manuscript. All authors approved the final version of the manuscript.
