# Supplementary material for: The effects of green tea supplementation on cardiovascular risk factors: A systematic review and meta-analysis
Source: Front Nutr. 2023 Jan 10;9:1084455. doi: 10.3389/fnut.2022.1084455 (PMC9871939; doi:10.3389/fnut.2022.1084455)
Supplement: Supplementary file 1 [file Table_2.DOCX]

A)

B)

C)

D)

E)

F)

G)

H)

I)

J)

K)

Supplementary figure 1. Funnel plots for the effects of green tea extract supplementation on A) TG (mg/dL); B) TC (mg/dL); C) LDL (mg/dL); D) HDL (mg/dL); E) FBS (mg/dL); F) fasting insulin (μlU/ml); G) HbA1c (%); H) HOMA-IR; I) SBP (mmHg), J) DBP (mmHg) and CRP (mg/dL).

A)

B)

C)

D)

E)

F)

G)

H)

I)

J)

K)

Supplementary figure 2. Non-linear dose-response relations between green tea extract supplementation and absolute mean differences. Dose-response relations between dose (g/day) and absolute mean differences in A) TG (mg/dL); B) TC (mg/dL); C) LDL (mg/dL); D) HDL (mg/dL); E) FBS (mg/dL); F) fasting insulin (μlU/ml); G) HbA1c (%); H) HOMA-IR; I) SBP (mmHg), J) DBP (mmHg) and CRP (mg/dL).

A)

B)

C)

D)

E)

F)

G)

H)

I)

J)

K)

Supplementary figure 3. Non-linear dose-response relations between green tea extract supplementation and absolute mean differences. Dose-response relations between duration of intervention (week) and absolute mean differences in A) TG (mg/dL); B) TC (mg/dL); C) LDL (mg/dL); D) HDL (mg/dL); E) FBS (mg/dL); F) fasting insulin (μlU/ml); G) HbA1c (%); H) HOMA-IR; I) SBP (mmHg), J) DBP (mmHg) and CRP (mg/dL).

A)

B)

C)

D)

E)

F)

G)

H)

I)

J)

K)

Supplementary figure 4. linear dose-response relations between green tea extract supplementation and absolute mean differences. Dose-response relations between dose (g/day) and absolute mean differences in A) TG (mg/dL); B) TC (mg/dL); C) LDL (mg/dL); D) HDL (mg/dL); E) FBS (mg/dL); F) fasting insulin (μlU/ml); G) HbA1c (%); H) HOMA-IR; I) SBP (mmHg), J) DBP (mmHg) and CRP (mg/dL).

A)

B)

C)

D)

E)

F)

G)

H)

I)

J)

K)

Supplementary figure 5. linear dose-response relations between green tea extract supplementation and absolute mean differences. Dose-response relations between duration of intervention (week) and absolute mean differences in A) TG (mg/dL); B) TC (mg/dL); C) LDL (mg/dL); D) HDL (mg/dL); E) FBS (mg/dL); F) fasting insulin (μlU/ml); G) HbA1c (%); H) HOMA-IR; I) SBP (mmHg), J) DBP (mmHg) and CRP (mg/dL).
