# Supplementary material for: The effects of green tea supplementation on cardiovascular risk factors: A systematic review and meta-analysis
Source: Front Nutr. 2023 Jan 10;9:1084455. doi: 10.3389/fnut.2022.1084455 (PMC9871939; doi:10.3389/fnut.2022.1084455)
Supplement: Supplementary file 2 [file Table_3.DOCX]

| terms | |
| --- | --- |
| "green tea" OR "green tea extract" OR "catechin" OR "catechins" OR "Camellia sinensis" OR "Thea sinensis" | Intervention OR "controlled trial" OR randomized OR randomised OR random OR randomly OR placebo OR "clinical trial" OR Trial OR "randomized controlled trial" OR "randomized clinical trial" OR RCT OR blinded OR "double blind" OR "double blinded" OR trial OR "clinical trial" OR trials OR "Cross-Over" OR parallel |

| terms | |  |
| --- | --- | --- |
| PubMed | ("green tea"[Title/Abstract] OR "green tea extract"[Title/Abstract] OR "catechin"[Title/Abstract] OR "catechins"[Title/Abstract] OR "Camellia sinensis"[Title/Abstract] OR "Thea sinensis"[Title/Abstract]) AND (Intervention[Title/Abstract] OR "controlled trial"[Title/Abstract] OR randomized[Title/Abstract] OR randomised[Title/Abstract] OR random[Title/Abstract] OR randomly[Title/Abstract] OR placebo[Title/Abstract] OR "clinical trial"[Title/Abstract] OR Trial[Title/Abstract] OR "randomized controlled trial"[Title/Abstract] OR "randomized clinical trial"[Title/Abstract] OR RCT[Title/Abstract] OR blinded[Title/Abstract] OR "double blind"[Title/Abstract] OR "double blinded"[Title/Abstract] OR trial[Title/Abstract] OR trials[Title/Abstract] OR "Cross-Over"[Title/Abstract] OR parallel[Title/Abstract] OR) | 2054 |
| Scopus | ( TITLE-ABS-KEY ( "green tea" OR "green tea extract" OR "catechin" OR "catechins" OR "Camellia sinensis" OR "Thea sinensis" ) AND TITLE-ABS-KEY ( intervention OR "controlled trial" OR randomized OR randomised OR random OR randomly OR placebo OR "clinical trial" OR trial OR "randomized controlled trial" OR "randomized clinical trial" OR rct OR blinded OR "double blind" OR "double blinded" OR trial OR "clinical trial" OR trials OR "Cross-Over" OR parallel ) ) | 5316 |
| Web of science | "green tea" OR "green tea extract" OR "catechin" OR "catechins" OR "Camellia sinensis" OR "Thea sinensis" (Topic) and Intervention OR "controlled trial" OR randomized OR randomised OR random OR randomly OR placebo OR "clinical trial" OR Trial OR "randomized controlled trial" OR "randomized clinical trial" OR RCT OR blinded OR "double blind" OR "double blinded" OR trial OR "clinical trial" OR trials OR "Cross-Over" OR parallel (Topic) | 3916 |
| All |  | 11286 |
| Duplicates |  | 3529 |
| Remained |  | 7757 |

May 2022
